# Is gender inequity a risk factor for men reporting poorer self-rated health in the United States?

**DOI:** 10.1371/journal.pone.0200332

**Published:** 2018-07-17

**Authors:** Shane A. Kavanagh, Julia M. Shelley, Christopher Stevenson

**Affiliations:** 1 Department of Public Health, School of Psychology and Public Health, La Trobe University, Bundoora, Victoria, Australia; 2 School of Health & Social Development, Faculty of Health, Deakin University, Geelong, Victoria, Australia; Columbia University, UNITED STATES

## Abstract

Theoretical approaches suggest that gender inequity increases men’s health risks. Previous findings from the United States support this contention, however only a small number of health outcomes have been explored. This study extends the range of health outcomes examined by using a cross-sectional, multilevel analysis to investigate whether measures of state-level gender inequity are predictors of men’s self-rated health. Data were derived primarily from the Behavioral Risk Factor Surveillance System and the full-case data set included 116,594 individuals nested within 50 states. Gender inequity was measured with nine variables: higher education, women’s reproductive rights, abortion provider access, elected office, management, business ownership, labour force participation, earnings and relative poverty. Covariates at the individual level were age, income, education, race/ethnicity, marital status and employment status. Covariates at the state level were income inequality and gross domestic product per capita. In fully adjusted models for all-age men the reproductive rights (OR 1.06 95% CI 1.01–1.11), abortion provider access (OR 1.11 95% CI 1.05–1.16) and earnings (OR 1.06 95% CI 1.02–1.12) measures all predicted an increased risk of men reporting poorer self-rated health for each 1 standard deviation increase in the gender inequity z-score. The most consistent effect was seen for the 65+ age group where the reproductive rights (OR 1.09 95% CI 1.03–1.16), abortion provider access (OR 1.15 95% CI 1.09–1.21), elected office (OR 1.06 95% CI 1.01–1.11) and earnings (OR 1.10 95% CI 1.04–1.16) measures all showed a significant effect. These findings provide evidence that some aspects of gender inequity increase the risk of poorer self-rated health in men. The study contributes to a growing body of literature implicating gender inequity in men’s health patterns.

## Introduction

For many important measures men have poorer health than women [1,2]. Men for example have higher mortality in almost all countries [3]. This pattern is borne out in the United States (US) where men have a 4.8-year lower life expectancy than women [4]. Men are also at a heightened risk of many of the most serious chronic diseases [1] with men in the US at greater risk of suffering from diabetes and coronary heart disease [5,6].

Biological differences between men and women are likely to contribute to these health differences [7]. However, the variability of men’s health relative to women across different social contexts suggests that social processes are of primary importance [2,8]. Gaining an understanding of these social processes is likely to make an important contribution to the public health literature.

Gender inequity is one such social process implicated in men’s health. It refers primarily to the systematic differences in political, economic and social power between women and men [9]. The multiple social, political and economic benefits that men receive from gender inequity [10] could be expected to contribute to men experiencing better health. However, recent theoretical developments suggest that gender inequity has the potential instead to shape the social environment in ways that damage men’s health.

An important approach to understanding the relationship between gender inequity and men’s health is masculinities and health theory [11]. It argues men’s poor health is a consequence of health behaviours tied to idealised gender norms [1,11–13]. These gender norms, emphasising attributes such as strength and invulnerability, provide the justification for power differences between men and women and also amongst men [11, 14]. In particular, a disregard for physical discomfort, risk-taking and a lack of attention to health are ways men assert their superiority to women and prove their ranking amongst ‘real’ men [11] p. 1390.

Masculinities and health theory argues these idealised norms, which support gender inequity, increase the likelihood men will engage in a range of poor health-related behaviours, such as smoking, over consumption of alcohol and poor dietary habits [11]. Further, the theory suggests that they may deter men from engaging with health services thus reducing the likelihood of identifying health issues in a timely manner and also reducing exposure to health education messages [11,15].

Empirical studies have found that aspects of masculinity are associated with poor health behaviours, poor health-related beliefs and health outcome measures [16–28]. For example, a study of preventive health care use in over one thousand older, mainly white men in the US found those with strong masculinity beliefs were half as likely to have received health care as those with more moderate masculinity beliefs. Further, the normal benefit of higher socioeconomic status was reversed in those holding strong masculine beliefs [29]. However, it is important to note that the empirical findings are quite complex with studies also suggesting some aspects of masculinity are associated with better health behaviours and health outcomes [20–22,24,28,30–35].

Gender inequity may also increase men’s health risks by limiting the number of social roles men take on. Barnett and Hyde [36] argue undertaking a greater number of social roles, such as spouse, parent, or employee, has beneficial effects on the health of both men and women [36]. The health benefits of expanded social roles are argued to arise through multiple pathways, but particularly important are psychological processes. For example, expanded roles for men in the household and in childcare may increase shared relationship experiences that facilitate greater communication and improved relationship quality [36]. Expanded roles may also increase opportunities for men to experience success leading to greater self-confidence and self-efficacy [36]. Additionally, they may facilitate the development of greater self-complexity and a greater frame of reference allowing men a broader perspective on successes and failures in specific areas of their lives [36]. These psychological factors may protect men’s health by providing a range of resources that act to buffer against the negative effects of stress and increase possibilities for social support [36]. For example, men who undertake household management and childcare may be able to access psychological and social resources that provide protection from threats to self-esteem that arise in the workplace [36].

Gender inequity may also be important for men’s health because it is tied to broader processes that impact on the social and economic resources that are available to men [37–39]. Improvements in women’s social position have been linked to greater investment in health related infrastructure and services as well as to better welfare provision [38, 40]. Such factors may help to protect against the health damaging effects of adverse economic, social and personal circumstances such as poverty and unemployment [37].

Gender inequity exists at multiple social levels. The measurement of gender inequity across larger social groups is important as gender inequity is sustained by social processes related to societal institutions [41]. Within the US states are important units of analysis. They represent administrative areas with distinct legal, political and socioeconomic cultures and policies. As such, they provide a unit of analysis that is potentially sensitive to the variance of gender inequity across US society. This is borne out by evidence demonstrating large differences in measures of gender inequity between US states [42].

Previous empirical studies from the US have provided evidence that state-level gender inequity increases men’s health risks. In an early study, Kawachi et al. [43] found that some state level measures of women’s status were associated with decreased mortality, but not with activity limitations. The authors explain the findings by suggesting women’s economic insecurity impacts on the material wellbeing of men in the household. Roberts [44] also found evidence that aspects of state-level gender equality decreased the likelihood of men engaging in riskier alcohol consumption as well as the volume of alcohol consumed. The findings at least partially support the author’s suggestion that gender equity may reduce men’s alcohol use by increasing their number of social roles and hence life satisfaction. Holter [45] identified that measures of gender equality at the state level were associated with greater wellbeing in men and women and a lower risk of violent death in men. The author highlights enhanced communication and changes in male roles as potential health enhancing effects of gender equality. Most recently, Kavanagh et al. [46] have shown that a number of measures of state-level gender inequity predicted higher mortality risk in men. The authors suggest the same theoretical approaches as outlined here.

One health measure that has not been examined is self-rated health (SRH). SRH is a strong predictor of mortality [47–50]. Understanding the relationship between gender inequity and men’s SRH would make an important contribution to answering the question of whether gender inequity increases men’s health risks.

An appropriate analytical approach for investigating the relationship between state-level gender inequity and men’s health is multilevel modelling. It allows for investigating group level, or contextual factors that contribute to the health of individuals [51–53]. The current study takes a multilevel modelling approach to examine the relationship between measures of state-level gender inequity and men’s SRH within the US.

## Methods

### Study sample

Individual level health and covariate data were taken from the Behavioral Risk Factor Surveillance System (BRFSS) 2005 data set [54]. The BRFSS is an annual survey established by the Centers for Disease Control and Prevention to collect data on behavioural risk factors and preventive health practices in household dwelling adults in US states and territories [55]. Data were collected via a telephone survey from a random sample of adults reflecting one adult per household [55]. The current study utilised data collected from the 50 US states.

### Outcome measure

The outcome measure was SRH. To measure SRH the BRFSS survey questionnaire asks respondents: Would you say that in general your health is excellent/very good/good/fair/poor? [54]. The current analysis used a dichotomised measure of excellent, very good or good SRH (0) versus fair or poor SRH (1).

### Gender inequity measures

Gender inequity was measured with relative measures of women’s social position because the use of absolute measures of women’s social position, such as women’s status, to infer gender inequity is potentially problematic [44]. For example, two areas with similar levels of educational attainment for women may have very different levels of men’s attainment [44]. An exception to the use of relative measures was made for measures related to reproductive issues. In cases where measures of gender inequity deal with sex-specific needs relative measures are not meaningful.

The analysis used nine state level measures of gender inequity. Three measures were taken from the Status of Women in the States (SWS) report produced by the Institute of Women’s Policy Research [42]. This report provides state level measures across a range of domains. The first was the women’s reproductive rights composite index. It combines a number of measures of access to reproductive services, including abortion, contraception and fertility treatment, as well as policies on second parent same-sex adoption and sex education in schools. The second measure was abortion provider access, the percentage of women who live in a county with at least one abortion provider. This measure is a component measure of the reproductive rights composite measure. The final measure taken from the SWS report was the elected office measure, a composite measure reflecting women’s office-holding at the state and national levels for each state.

The remaining measures were calculated from US Census Bureau data. These measures were higher education, the proportion of men with a Bachelor’s degree or higher divided by the proportion of women with a Bachelor’s degree or higher expressed as a percentage; management, the proportion of men employed in management occupations divided by the proportion of women in management occupations expressed as a percentage; business ownership, the number of male-owned businesses divided by the number of female or equally owned businesses expressed as a percentage; labour force participation, the percentage of men in the labour force divided by the percentage of women in the labour force expressed as a percentage; earnings, the median earnings of full-time, year-round employed men divided by those of women expressed as a percentage; and relative poverty, the proportion of females below poverty divided by proportion of males below poverty expressed as a percentage.

The gender inequity measures were converted to *z*-scores to allow comparability. For all measures increasing value signifies increasing inequity. In the case of the reproductive rights, abortion provider and elected office measures, this was achieved by multiplying the *z*-score by -1.

### Covariates

Individual-level covariates included age in years (18–99 years); income as measured by equivalised household income, a continuous variable calculated from the eight-category household income measure: less than $10,000; $10,000 to <$15,000; $15,000 to <$20,000; $20,000 to <$25,000; $25,000 to <$35,000; $35,000 to <$50,000; $50,000 to <$75,000; $75,000 or more. The mid-point for each category was divided by the square root of the total number of persons in each household [56]. The value of the top category was calculated using a Pareto distribution [57]. Values were standardised to a *z*-score. One concern with this measure was that the value for the top code calculated using a Pareto distribution was $286,319. This appeared excessive. To deal with this concern sensitivity testing included modelling the income variable in the form of the initial categories.

Further individual level covariates included: Education as measured with four categories: did not graduate high school (*reference*), graduated high school, attended college or technical school, and graduated from college or technical school. Race and ethnicity as measured with five categories: white-non-Hispanic (*reference*), black-non-Hispanic, other race only-non-Hispanic, multiracial-non-Hispanic, Hispanic. Marital status, which was coded to two categories: married or member of an unmarried couple (*reference*) versus those who were divorced, widowed, separated or never married. Employment status, which was coded to two categories: other (*reference*) including employed, self-employed, homemaker, student, retired and unable to work versus unemployed.

The state level covariates were income inequality and area-level socioeconomic position. Income inequality was measured by the Gini coefficient. The Gini coefficient is a value between 0 and 1 with 0 representing perfect equality where all entities, such as individuals or households, have equal income and 1 representing perfect inequality where one entity has all income [58]. Values were derived from US Census Bureau data and measure household income inequality.

State area-level socioeconomic position was measured by per capita gross domestic product (GDP). Per capita GDP is a measure of average standard of living or economic wellbeing [59]. Values were derived from the US Department of Commerce, Bureau of Economic Analysis. A second measure, state median household income, was also included for sensitivity testing as this is also a commonly used measure of area-level socioeconomic position. It reflects the three-year average from 2002 to 2004 in 2004 dollars and was taken from the US Census Bureau. Both measures were standardised to *z*-scores to allow comparability with the gender inequity measures.

### Missing data

The full data set contained 131,879 cases nested in 50 states. A full-case analysis led to the loss of approximately 12% of cases. This arose primarily due to missing data for income in approximately 10% of cases. For other variables missing data were minimal (< = 1%). To deal with concerns of bias raised by this missing data several models were re-estimated with multiple imputation of missing income data in sensitivity analysis. This was undertaken with the REALCOM Impute software program [60], which allows multiple imputation in two-level data sets. Estimation of a complex model with a large number of cases proved extremely computationally intensive. As such, a work-around was used for modelling the effects of missing data. This involved creating smaller data sets restricted by age (65+ years and 40–49 years) with missing data only on the variable of primary concern: income.

### Analysis

The full-case data set consisted of 116,594 individuals nested within 50 states. Initial analysis of the data explored correlations between state level variables. Correlation coefficients were calculated with SPSS software version 21 [61] utilising the bivariate Pearson two-tailed analysis.

Multilevel logistic regression was undertaken with MLwiN version 2.31 [62]. An initial null model was estimated to ascertain the existence of significant state-level variance. Subsequently, a separate model was estimated for each gender inequity measure. The model was specified as:

$$\begin{array}{ll} logit(srh_{ij})= & cons+age_{ij}+income_{ij}+education2_{ij}+education3_{ij}+education4_{ij}\\ & +race/ethnicity2_{ij}+race/ethnicity3_{ij}+race/ethnicity4_{ij}\\ & +race/ethnicity5_{ij}+employment\;status2_{ij}+marital\;status2_{ij}\\ & +Gini_{j}+GDP_{j}+gender\;inequity\;measure_{j} \end{array}$$

Categorical variables were fitted so that their coefficients represent log odds ratios with reference to category 1. Continuous variables were grand-mean centred in modelling. Age restricted models were also estimated for the 18–64 years and 65+ age groups as initial testing suggested age-specific effects.

Estimation was undertaken using a second order penalised quasi-likelihood (PQL2) approach. PQL2 estimation provides the least biased estimates of a number of quasi- likelihood methods and does not entail the large computational requirements of Markov Chain Monte Carlo (MCMC) or Bootstrapping techniques [63–65]. However, in some cases, PQL2 estimation may lead to biased results in comparison to these techniques [64]. To overcome such concerns sensitivity testing included re-estimation of one of the models with the MCMC estimator [66] to check for potential bias [65]. The MCMC settings were a burn-in of 5000 and 100,000 iterations.

Ethics exemption was provided by the Deakin University Human Research Ethics Committee (reference: 2013–022).

## Results

Descriptive statistics are provided in Table 1. There is a consistent pattern of benefits in favour of men for all state-level gender inequity measures. However, it should be noted that in the case of the higher education measure some states did not follow the overall trend. In four states the level of the male population attaining four or more years of college was less than that for women: Alaska (87.53%), Kentucky (99.04%), South Dakota (98.20%) and Vermont (88.99%).

**Table 1:** Descriptive statistics.

Measure	Categories	Range (min)	Range (max)	Mean	SD	N	%
***Individual level (n = 116594)***							
SRH	Good, very good, excellent health (0)					96775	83
	Fair or poor health (1)					19819	17
Age (yrs)		18	99	50.71	16.22		
Income (equivalised)		1212.68	286319	66128	73713		
(zscore)		-0.88	2.99	0	1		
Education	Did not graduate high school (1 reference)					11242	9.6
	Graduated high school (2)					34743	29.8
	Attended college or technical school (3)					29199	25
	Graduated college or technical school (4)					41410	35.5
Race	White—non Hispanic (1 reference)					95439	81.9
	Black—non Hispanic (2)					6725	5.8
	Other race only—non Hispanic (3)					5037	4.3
	Multiracial—non Hispanic (4)					2266	1.9
	Hispanic (5)					7127	6.1
Unemployment	other (1 reference)					112246	96.3
	unemployed (2)					4348	3.7
Marital status	Married/member of an unmarried couple (1 ref.)					75973	65.2
	Divorced/widowed/separated/never married (2)					40621	34.8
***State Level (n = 50)***							
Higher education (%)		87.53	127.94	107.48	6.66		
(zscore)		-3.00	3.07	0	1		
Reproductive rights*		0.27	6.25	2.78	1.71		
(zscore)		-1.47	2.03	0	1		
Abortion provider*		12	100	56.42	24.42		
(zscore)		-1.82	1.78	0	1		
Elected office*		0.64	4.38	2.11	0.88		
(zscore)		-1.66	2.58	0	1		
Management (%)		112.63	221.55	154.37	22.41		
(zscore)		-1.86	3.00	0	1		
Business ownership (%)		108.13	177.71	141.44	18.12		
(zscore)		-1.84	2	0	1		
Labour force (%)		108.87	128.51	117.67	4.24		
(zscore)		-2.07	2.56	0	1		
Earnings (%)		121.39	164.53	133.30	7.53		
(zscore)		-1.58	4.15	0	1		
Relative poverty (%)		104.90	141.38	127.07	6.55		
(zscore)		-3.38	2.18	0	1		
GDP (per capita)		30750	65541	45402.38	7724.133		
(zscore)		-1.90	2.61	0	1		
Gini		0.39	0.49	0.44	0.02		
(zscore)		-2.44	2.38	0	1		

*z-score multiplied by -1 so that increasing value represents increasing gender inequity

The results of the correlation analysis show a high number of statistically significant correlations between the gender inequity measures (see Table 2). These were all positive, with the exception of the correlation between labour force and management, which was negative (r = –0.40). The strongest correlation was between the reproductive rights composite measure and the abortion provider measure (r = 0.73). This was expected, as the abortion provider measure is a component of the reproductive rights composite measure. These two measures in turn had moderate positive correlations with the elected office, management and earnings measures (between r = 0.41 and r = 0.59). A further moderate positive correlation was noted between business ownership and relative poverty (r = 0.45). The higher education measure was moderately positively correlated with the labour force measure (r = 0.60).

**Table 2:** State level correlations.

	**Higher educ**.	**Rep. rights**	**Provider**	**Elected off**.	**Managem**.	**Bus. owner**.	**Lab. force**	**Earnings**	**Rel. poverty**	**Gini**	**GDP**
**Higher education**	1	0.14	-0.14	-0.00	0.02	0.13	0.60**	-0.02	0.29*	0.13	-0.13
**Reproductive rights**		1	0.73**	0.45**	0.42**	0.05	0.02	0.41**	0.16	-0.14	-0.54**
**Abortion provider**			1	0.49**	0.43**	-0.03	-0.18	0.59**	0.23	-0.21	-0.59**
**Elected office**				1	0.26	0.38**	-0.04	0.18	0.28*	-0.02	-0.37**
**Management**					1	-0.01	-.40**	0.34*	0.22	-0.36*	-0.27
**Business ownership**						1	-0.02	-0.06	0.45**	0.41**	0.07
**Labour force**							1	-0.14	-0.18	0.31*	-0.09
**Earnings**								1	0.23	-0.37**	-0.17
**Relative poverty**									1	0.12	-0.04
**Gini**										1	-0.01
**GDP**											1

* Significant at the 0.05 level (2-tailed); Pearson Bivariate (n = 50)

** Significant at the 0.01 level (2-tailed);

The state level covariates of GDP and income inequality were, in some cases, correlated with the gender inequity measures. The GDP measure was negatively correlated with the reproductive rights (r = –0.54), abortion provider (r = –0.59) and the elected office (r = –0.37) measures. The Gini showed a more mixed pattern of correlation being positively correlated with the business ownership (r = 0.41) and labour force (r = 0.31) measures and negatively correlated with the management (r = –0.36) and the earnings (r = –0.37) measures. The negative correlation between the Gini and gender inequity in earnings is unexpected.

The results of the logistic multilevel null model gave a state-level variance of 0.069 (s.e. 0.015), which is highly significant [65]. The intraclass correlation coefficient (ICC) was estimated at 0.021. This suggests 2.1% of the variance of SRH is due to between state variance. However, the ICC in logistic models is problematic and should be interpreted with caution [67].

**Fig 1:**
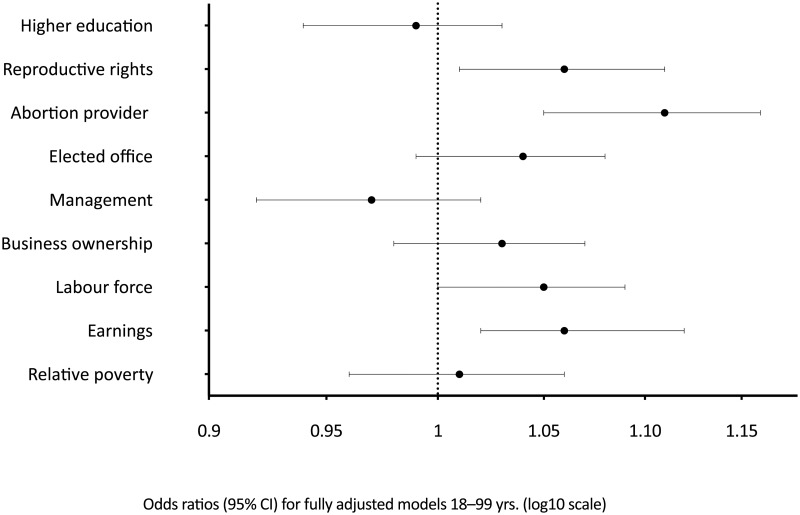
Odds ratios (95% CI) for reporting poor/fair versus good/very good/excellent SRH for one standard deviation increase in gender inequity *z*-score in multilevel logistic regression for 18–99 yrs.

The results of the full regression models for the gender inequity measures and SRH were mixed (see Table 3). Fig 1 provides a graph of the results for the 18–99 age group. A number of measures showed no statistically significant association with SRH: higher education, management, business ownership and relative poverty. However, several measures did show a significant association. Of these the strongest and most consistent was the abortion provider measure. It was positively associated in all age ranges. The association was strongest for the 65+ age group with an odds ratio of 1.15 for each one standard deviation increase in the standardised score.

**Table 3:** Odds ratios (95% CI) for reporting poor/fair versus good/very good/excellent SRH for one standard deviation increase in gender inequity *z*-score in multilevel logistic regression (see Tables A-C in S1 File for full regression results).

	18–99 yrs.	18–64 yrs.	65+ yrs.
**Gender inequity measure**			
**Higher education**	0.99 (0.94–1.03)	1.00 (0.95–1.05)	0.98 (0.93–1.03)
**Reproductive rights**	1.06* **(1.01–1.11)**	1.04 (0.98–1.10)	1.09* **(1.03–1.16)**
**Abortion provider**	1.11* **(1.05–1.16)**	1.08* **(1.02–1.14)**	1.15* **(1.09–1.21)**
**Elected office**	1.04 (0.99–1.08)	1.03 (0.98–1.08)	1.06* **(1.01–1.11)**
**Management**	0.97 (0.92–1.02)	0.96 (0.91–1.02)	1.01 (0.95–1.07)
**Business ownership**	1.03 (0.98–1.07)	1.03 (0.98–1.08)	1.02 (0.97–1.07)
**Labour force**	1.05 (1.00–1.09)	1.07* **(1.02–1.12)**	1.01 (0.96–1.07)
**Earnings**	1.06* **(1.02–1.12)**	1.04 (0.99–1.10)	1.10* **(1.04–1.16)**
**Relative poverty**	1.01 (0.96–1.06)	1.01 (0.96–1.05)	1.03 (0.98–1.09)

*sig. (β/s.e. >2)

Odds ratios in models adjusted for age, income, education, race/ethnicity, employment status, marital status, Gini and GDP

Among the other measures the pattern of association was dependent on the age range modelled. For example, the reproductive rights measure was not significantly associated in the 18–64 age group, but was for the 18–99 and 65+ age groups. The elected office measure was only significant in the 65+ age group. The labour force measure in contrast was only significant in the 18–64 year age group. Last, the earnings measure was significantly associated in the 18–99 and 65+ age groups.

The effect sizes for the gender inequity measures were generally smaller than those seen for the income inequality variable. In many cases they also showed a weaker effect than the state-level socioeconomic position variable. However, in other cases they showed a similar or stronger effect (see Tables A-C in S1 File).

Sensitivity testing with the income variable in its original, non-equivalised categorical form led to only small changes in the coefficient values for the gender inequity variables (see Table A in S2 File). The substitution of state median household income for state GDP also generally only caused small changes in the gender inequity SRH associations (see Table B in S2 File). These changes did however lead to the reproductive rights and earnings coefficients becoming marginally non-significant, while the labour force coefficient became significant. Sensitivity testing with MCMC estimation led to only minor changes in the re-estimated reproductive rights model (see Table C in S2 File).

Finally, analysis of subsets of data with multiple imputation of missing income data also led to only minor changes in the estimates. This suggests that a full-case analysis was not likely to have biased the results (see Table D in S2 File).

## Discussion

The results of this study suggest that some aspects of state-level gender inequity increase the risk of men reporting poorer self-rated health. The most consistent effect was seen for the reproductive health measures: reproductive rights and abortion provider access. In particular, greater inequity in the abortion provider measure was associated with poorer SRH in all of the age ranges with the strongest association seen for the oldest age group. The same effect was also seen for the elected office measure in older men, the labour force measure in working-age men and the earnings measure in all-age and older men. However, a number of the gender inequity measures showed no statistically significant association with SRH in any of the models: higher education, management, business ownership and relative poverty. These results suggest that only some aspects of gender inequity increase men’s risk of reporting poorer SRH.

The effect sizes are small. For example, in the 65+ age group the elected office measure was associated with a 16% increase in risk for the highest gender inequity state compared to the lowest. In the case of the abortion provider measure the risk increase was 36% (calculations not shown). These small effects however have important population health implications as they represent the impact of a population-wide exposure on a commonly occurring measure of poorer health [68].

Several aspects of the findings deserve particular consideration. First, the relationship between measures of gender inequity and SRH appear to be age dependent. In most cases the strongest associations were noted in the older age group. The pattern of stronger effects at older ages is unexpected given that health effects are often less obvious in older age groups due to the higher background level of poor health [69]. The reasons for this finding are not clear. There may be a cohort effect involving older age groups. Alternatively, it is possible that some gender inequity measures are markers for other social processes that impact disproportionately at older ages.

A further important finding is that gender inequity in earnings was a significant or borderline significant predictor of poorer SRH in all of the models. Importantly, the Gini coefficient measure was also statistically significant in each of these models (see Tables A-C in S1 File). This suggests that the gender gap in earnings may have an independent negative impact on the health of men in addition to that of household income inequality. Such a finding makes an interesting contribution to the literature regarding income inequality and health. Pickett and Wilkinson [70] have argued that greater income inequality is a strong driver of poor health patterns in developed countries. Income inequality measures that are sensitive to gender differences in earnings could potentially reveal stronger and more consistent effects.

It is also informative to compare the present findings with those from a previous multilevel study using almost identical measures of state-level gender inequity to examine the effects of gender inequity on men’s mortality in the US [46]. With the exception of the earnings measure, the measures that were predictors of SRH in this study were different from the measures that were predictors of men’s mortality in the earlier study. This may indicate different pathways linking gender inequity to men’s SRH than those linking to mortality. It may also be the result of the studies occurring in different time periods with the previous study examining data from the 1990’s.

From a broad perspective the findings of this study support those from previous studies in the US. As discussed above, studies have found an association between some state level measures of gender inequity, or related concepts, and poorer health outcomes and health behaviours in men. Of particular relevance, given the strong link between SRH and mortality, are previous studies investigating the relationship between measures of women’s social position and mortality. Specifically, Kawachi et al. [43] and Kavanagh et al. [46] have found that women’s status and gender inequity predict men’s mortality risk. Holter [45] also found that measures of gender equality at the state level predicted lower risk of violent death in men. Taken together with the current study, these findings suggest that aspects of gender inequity, at least when measured at the state level, may be important factors in explaining men’s mortality patterns in the US.

The increasing evidence that aspects of gender inequity are predictive of poorer health for men in the US suggests the possibility of a causal relationship. The theoretical approaches discussed in the introduction to this paper suggest plausible pathways. Gender inequity may be related to masculine ideals that increase the risk of men engaging in poor health behaviours. It may also be related to a limitation of the number of social roles that men can engage in leading to a loss of the social and psychological benefits of multiple roles. Further still, the extent of gender inequity may impact on men’s health because it is related to broader investments in social resources available to men in times of need. The current study was not able to provide any evidence to support one or other of these particular pathways. Future work will be required that tests specific pathways before further assertions can be made regarding the processes linking gender inequity to the health of men.

The findings of this study reinforce the importance of addressing gender inequity in order to improve the health of societies. Here, we note previous work undertaken for the World Health Organization, which provides approaches that address the issue of gender inequity as a social determinant of health with a view to benefitting the health of both women and men [9].

The study has a number of strengths. First, it took a multilevel approach allowing exploration of the association between gender inequity measured at the social level and individual level SRH. Second, the data are from a large, representative data set increasing the generalisability of the findings to the population of interest. Third, a wide range of measures of gender inequity was used. Importantly, these measures focussed on relative differences with the exception of the reproductive measures. Last, the study controlled for a large number of potential individual and state level confounders.

The study also has a number of limitations. First, the cross-sectional design limits causal inferences. A second limitation is that the gender inequity variables were measured at one social level. While some measures of gender inequity may be best captured at the state level, others may not be. For example, the abortion provider measure of percentage of women living in a county with a provider may be best modelled at the county level. Further, measures such as labour force participation may have different magnitudes and impacts on men’s health when measured at the household level.

A third limitation is the limited gradations of some of the categorical covariates. For example, the measurement of household income was limited by a lack of gradation for the upper segment of the income spectrum with a top category of >$75,000. Similar concerns exist with regards to the measurement of education where the highest category had the largest number of cases. These measurement limitations leave open the possibility of residual confounding. A final limitation is that income was measured at the household level leaving open the possibility of confounding by household size. While this issue was partly overcome by calculation of an equivalised income measure, this represents an approximation. It is unclear what impacts the above noted socioeconomic measurement issues are likely to have had on the results. However, given that multiple different measures of socioeconomic position were included in the modelling, the impacts are likely to have been relatively small.

## Conclusion

Gender inequity is a pervasive influence on society. This study provides evidence that aspects of state-level gender inequity predict poorer SRH for men in the US. It contributes to a growing body of literature that suggests that, as well as impacting on women, gender inequity may contribute to the poor health of men.

## Supporting information

S1 File Regression Tables A-C. (PDF)

S2 File Sensitivity testing Tables A-D. (PDF)

S3 File Notes on data preparation. (PDF)

## References

[1] Courtenay W. Key determinants of the health and well-being of men and boys. An overview In: Courtenay W, *Dying to be men*: *psychosocial*, *environmental*, *and biobehavioral directions in promoting the health of men and boys* [Internet]. Hoboken: Taylor and Francis; 2011. Available: ProQuest Ebook Central

[2] European Union. The state of men’s health in Europe [Internet]. Directorate-General for Health and Consumers, European Commission; 2011. http://ec.europa.eu/health/population_groups/docs/men_health_extended_en.pdf

[3] Wang H, Dwyer-Lindgren L, Lofgren KT, Rajaratnam JK, Marcus JR, Levin-Rector A, et al Age-specific and sex-specific mortality in 187 countries, 1970–2010: a systematic analysis for the Global Burden of Disease Study 2010. The Lancet. 2012; 380: 2071–2094. 10.1016/S0140-6736(12)61719-X 23245603

[4] Kochanek KD, Murphy SL, Xu JQ. Deaths: final data for 2011. National vital statistics reports. Natl Cent Health Stat. 2015; 63 https://www.cdc.gov/nchs/data/nvsr/nvsr63/nvsr63_03.pdf 26222597

[5] Centers for Disease Control and Prevention. Prevalence of coronary heart disease—United States, 2006–2010 morbidity and mortality weekly report. 2011; 60: 1377–1381. https://www.cdc.gov/mmwr/pdf/wk/mm6040.pdf 21993341

[6] Centers for Disease Control and Prevention. Age-adjusted rates of diagnosed diabetes per 100 civilian, non-institutionalized population, by sex, United States, 1980–2014 [Internet]. 2015 [cited 10 November 2016]. http://www.cdc.gov/diabetes/statistics/prev/national/figbysex.htm.

[7] Seifarth JE, McGowan CL, Milne KJ. Sex and life expectancy. Gend Med. 2012; 9: 390–401. 10.1016/j.genm.2012.10.001 23164528

[8] Luy M, Gast K. Do women live longer or do men die earlier? Reflections on the causes of sex differences in life expectancy. Gerontology. 2014; 60: 143–153. 10.1159/000355310 24296637

[9] Sen G, Östlin P. Unequal, unfair, ineffective and inefficient gender inequity in health: Why it exists and how we can change it [Internet]. Women and Gender Equity Knowledge Network; 2007. http://www.who.int/social_determinants/resources/csdh_media/wgekn_final_report_07.pdf

[10] World Economic Forum. Global gender gap report 2016 [Internet]. World Economic Forum; 2016. http://www3.weforum.org/docs/GGGR16/WEF_Global_Gender_Gap_Report_2016.pdf

[11] Courtenay WH. Constructions of masculinity and their influence on men’s well-being: a theory of gender and health. Soc Sci Med. 2000; 50: 1385–1401. 10.1016/S0277-9536(99)00390-1 10741575

[12] Evans J, Frank B, Oliffe JL, Gregory D. Health, illness, men and masculinities (HIMM): a theoretical framework for understanding men and their health. J Mens Health. 2011; 8: 7–15. 10.1016/j.jomh.2010.09.227

[13] Pyke KD. Class-based masculinities: the interdependence of gender, class, and interpersonal power. Gend Soc. 1996; 10: 527–549.

[14] Connell RW, Messerschmidt JW. Hegemonic masculinity rethinking the concept. Gend Soc. 2005; 19: 829–859.

[15] Courtenay W. Key determinants of the health and well-being of men and boys. Int J Mens Health. 2003; 2: 1–30. 10.3149/jmh.0201.1

[16] Blazina C, Watkins CEJ. Masculine gender role conflict: Effects on college men’s psychological well-being, chemical substance usage, and attitudes towards help-seeking. J Couns Psychol. 1996;43: 461–465. 10.1037/0022-0167.43.4.461

[17] Courtenay W, McCreary DR. Masculinity and gender role conflict How they influence the likelihood that men will engage in multiple high-risk behaviors In: Courtenay W, *Dying to be Men*: *Psychosocial*, *environmental*, *and biobehavioral directions in promoting the health of men and boys*. Hoboken: Taylor and Francis; 2011.

[18] Dworkin SL, Treves-Kagan S, Lippman SA. Gender-transformative interventions to reduce HIV risks and violence with heterosexually-active men: A review of the global evidence. AIDS Behav. 2013;17: 2845–2863. 10.1007/s10461-013-0565-2 23934267

[19] Galdas PM, Cheater F, Marshall P. Men and health help-seeking behaviour: literature review. J Adv Nurs. 2005;49: 616–623. 10.1111/j.1365-2648.2004.03331.x 15737222

[20] Iwamoto DK, Liao L, Liu WM. Masculine norms, avoidant coping, Asian values, and depression among Asian American men. Psychol Men Masculinity. 2010;11: 15–24. 10.1037/a0017874 20657794 PMC2906828

[21] Iwamoto DK, Corbin W, Lejuez C, MacPherson L. College men and alcohol use: Positive alcohol expectancies as a mediator between distinct masculine norms and alcohol use. Psychol Men Masculinity. 2014;15: 29–39. 10.1037/a0031594 25705133 PMC4334455

[22] Levant RF, Wimer DJ. Masculinity constructs as protective buffers and risk factors for men’s health. Am J Mens Health. 2014;8: 110–120. 10.1177/1557988313494408 23832955

[23] Mahalik JR, Burns SM, Syzdek M. Masculinity and perceived normative health behaviors as predictors of men’s health behaviors. Soc Sci Med. 2007;64: 2201–2209. 10.1016/j.socscimed.2007.02.035 17383784

[24] McCreary DR, Newcomb MD, Sadava SW. The male role, alcohol use, and alcohol problems: A structural modeling examination in adult women and men. J Couns Psychol. 1999;46: 109–124. 10.1037/0022-0167.46.1.109

[25] Noar SM, Morokoff PJ. The relationship between masculinity ideology, condom attitudes, and condom use stage of change: A structural equation modeling approach. Int J Mens Health. 2002;1: 43–58.

[26] Pirkis J, Spittal MJ, Keogh L, Mousaferiadis T, Currier D. Masculinity and suicidal thinking. Soc Psychiatry Psychiatr Epidemiol. 2017;52: 319–327. 10.1007/s00127-016-1324-2 28025691

[27] Rogers AA, DeLay D, Martin CL. Traditional masculinity during the middle school transition: associations with depressive symptoms and academic engagement. J Youth Adolesc. 2016; 1–16. 10.1007/s10964-016-0545-8 27435597

[28] Sloan C, Conner M, Gough B. How does masculinity impact on health? A quantitative study of masculinity and health behavior in a sample of UK men and women. Psychol Men Masculinity. 2014; 10.1037/a0037261

[29] Springer KW, Mouzon DM. “Macho men” and preventive health care: Implications for older men in different social classes. J Health Soc Behav. 2011;52: 212–227. 10.1177/0022146510393972 21490311

[30] de Visser RO, McDonnell EJ. “Man points”: masculine capital and young men’s health. Health Psychol Off J Div Health Psychol Am Psychol Assoc. 2013;32: 5–14. 10.1037/a0029045 22888820

[31] Hunt K, Sweeting H, Keoghan M, Platt S. Sex, gender role orientation, gender role attitudes and suicidal thoughts in three generations. Soc Psychiatry Psychiatr Epidemiol. 2006;41: 641–647. 10.1007/s00127-006-0074-y 16732400

[32] Månsdotter A, Lundin A, Falkstedt D, Hemmingsson T. The association between masculinity rank and mortality patterns: a prospective study based on the Swedish 1969 conscript cohort. J Epidemiol Community Health. 2009;63: 408–413. 10.1136/jech.2008.082628 19366891

[33] Månsdotter A, Lundin A. How do masculinity, paternity leave, and mortality associate?–A study of fathers in the Swedish parental & child cohort of 1988/89. Soc Sci Med. 2010;71: 576–583. 10.1016/j.socscimed.2010.05.008 20538394

[34] Sloan C, Gough B, Conner M. Healthy masculinities? How ostensibly healthy men talk about lifestyle, health and gender. Psychol Health. 2010;25: 783–803. 10.1080/08870440902883204 20204942

[35] Wade JC. Traditional masculinity and African American men’s health-related attitudes and behaviors. Am J Mens Health. 2009;3: 165–172. 10.1177/1557988308320180 19477729

[36] Barnett RC, Hyde JS. Women, men, work, and family. Am Psychol. 2001; 56: 781–796. 10.1037/0003-066X.56.10.781 11675985

[37] Clark R, Peck BM. Examining the gender gap in life expectancy: a cross-national analysis, 1980–2005. Soc Sci Q. 2012; 93: 820–837. 10.1111/j.1540-6237.2012.00881.x

[38] Young FW. Structural pluralism and life expectancy in less-developed countries: the role of women’s status. Soc Indic Res. 2001; 55: 223–240. 10.1023/A:1010982822560

[39] Young FW. The structural ecology of health and community [Internet]. Ithaca, NY: The Internet-First University Press; 2009. http://dspace.library.cornell.edu/bitstream/1813/11809/1/Young%20Structural%20Ecology%20Health%20and%20Community.pdf

[40] Bolzehndahl C, Brooks C. Women’s political representation and welfare state spending in 12 capitalist democracies. Soc Forces. 2007; 85: 1509–1534.

[41] Walby S. Theorising patriarchy. Sociology. 1989; 23: 213–234.

[42] Institute for Women’s Policy Research. Status of women in the states. Washington, DC, USA: Library of Congress; 2004.

[43] Kawachi I, Kennedy BP, Gupta V, Prothrow-Stith D. Women’s status and the health of women and men: a view from the States. Soc Sci Med. 1999; 48: 21–32. 10.1016/S0277-9536(98)00286-X 10048835

[44] Roberts SCM. Macro-level gender equality and alcohol consumption: A multi-level analysis across U.S. States. Soc Sci Med. 2012; 75: 60–68. 10.1016/j.socscimed.2012.02.017 22521679 PMC4086912

[45] Holter ØG. “What’s in it for men?” Old question, new data. Men Masculinities. 2014; 17: 515–548. 10.1177/1097184X14558237

[46] Kavanagh SA, Shelley JM, Stevenson C. Does gender inequity increase men’s mortality risk in the United States? A multilevel analysis of data from the National Longitudinal Mortality Study. SSM—Popul Health. 2017; 3: 358–365. 10.1016/j.ssmph.2017.03.003 29349229 PMC5769061

[47] Idler EL, Benyamini Y. Self-rated health and mortality: a review of twenty-seven community studies. J Health Soc Behav. 1997; 38: 21–37. 9097506

[48] Benyamini Y, Idler EL. Community studies reporting association between self-rated health and mortality additional studies, 1995 to 1998. Res Aging. 1999; 21: 392–401. 10.1177/0164027599213002

[49] Benyamini Y, Blumstein T, Murad H, Lerner-Geva L. Changes over time from baseline poor self-rated health: for whom does poor self-rated health not predict mortality? Psychol Health. 2011; 26: 1446–1462. 10.1080/08870446.2011.559231 22011289

[50] DeSalvo KB, Bloser N, Reynolds K, He J, Muntner P. Mortality prediction with a single general self-rated health question. A meta-analysis. J Gen Intern Med. 2006; 21: 267–275. 10.1111/j.1525-1497.2005.00291.x 16336622 PMC1828094

[51] Hox J. *Multilevel analysis*: *techniques and applications*. Second edition Taylor and Francis; 2010.

[52] Snijders TAB, Bosker RJ. *Multilevel analysis*: *an introduction to basic and advanced multilevel modeling*. Second Edition London: Sage Publications; 2012.

[53] Subramanian SV, Jones K, Duncan C. Multilevel methods for public health research [Internet]. In: Kawachi I, Berkman LF, editors. *Neighborhoods and health*. Oxford University Press; 2009 http://www.oxfordscholarship.com

[54] Centers for Disease Control and Prevention. Behavioral Risk Factor Surveillance System 2005 codebook report [Internet]. Centers for Disease Control and Prevention; 2007. http://ftp.cdc.gov/pub/data/brfss/Codebook_05.rtf

[55] Centers for Disease Control and Prevention. Behavioral Risk Factor Surveillance System Overview 2005 [Internet]. Centers for Disease Control and Prevention; 2014. https://www.cdc.gov/brfss/annual_data/2005/pdf/overview_05.pdf

[56] OECD. What are equivalence scales [Internet]. OECD. [cited 2 June 2017]. http://www.oecd.org/eco/growth/OECD-Note-EquivalenceScales.pdf

[57] Ligon E. The development and use of a consistent income measure for the general social survey. GSS Methodological, Report No. 64; 1994.

[58] Atkinson AB. *The economics of inequality*. Second Edition Oxford: Clarendon Press; 1983.

[59] OECD. GDP per capita in National accounts at a glance 2009 [Internet]. OECD Publishing; 2010. 10.1787/9789264075108-5-en

[60] Carpenter JR, Goldstein H, Kenward MG. REALCOM-IMPUTE software for multilevel multiple imputation with mixed response types. J Stat Softw. 2011; 45 Available: http://www.jstatsoft.org/v45/i05/paper

[61] IBM. IBM SPSS software [Internet]. 2015 [cited 11 Jun 2015]. http://www-01.ibm.com/software/analytics/spss/

[62] Rasbash J, Charlton C, Browne WJ, Healy M, Cameron B. MLwiN version 2.10, Centre for Multilevel Modelling, University of Bristol. 2009.

[63] Goldstein H, Rasbash J. Improved approximations for multilevel models with binary responses. J R Stat Soc Ser A Stat Soc. 1996; 159: 505–513. 10.2307/2983328

[64] Rodriguez G, Goldman N. Improved estimation procedures for multilevel models with binary response: a case-study. J R Stat Soc Ser A Stat Soc. 2001; 164: 339–355.

[65] Steele F. Learning environment for multilevel methodology and applications: module 7: multilevel models for binary responses [Internet]. Centre for Multi-Level Modelling University of Bristol; 2009. http://www.cmm.bris.ac.uk/lemma/

[66] Browne WJ. MCMC estimation in MLwiN version 3.00 Centre for Multilevel Modelling, Univeristy of Bristol. 2017.

[67] Merlo J, Chaix B, Ohlsson H, Beckman A, Johnell K, Hjerpe P, et al A brief conceptual tutorial of multilevel analysis in social epidemiology: using measures of clustering in multilevel logistic regression to investigate contextual phenomena. J Epidemiol Community Health. 2006;60: 290–297. 10.1136/jech.2004.029454 16537344 PMC2566165

[68] Bentley R, Kavanagh A. Looking at contextual effects through rose-coloured glasses: interpreting contextual effects in multilevel models of health. Australas Epidemiol. 2007; 14: 4–5.

[69] Subramanian S, Kawachi I. Commentary: Chasing the elusive null-the story of income inequality and health. Int J Epidemiol. 2007; 36: 596–9. 10.1093/ije/dym102 17557781

[70] Pickett KE, Wilkinson RG. Income inequality and health: a causal review. Soc Sci Med. 2015; 128: 316–326 10.1016/j.socscimed.2014.12.031 25577953

