# Supplementary material for: Is gender inequity a risk factor for men reporting poorer self-rated health in the United States?
Source: PLoS One. 2018 Jul 17;13(7):e0200332. doi: 10.1371/journal.pone.0200332 (PMC6049919; doi:10.1371/journal.pone.0200332)
Supplement: S1 File — (PDF) [file pone.0200332.s001.pdf]

Supporting information 1 - Table A: Multilevel logistic regression for SRH (18+ yrs.)

	Higher Edu.	S.E.	Rep. Rights	S.E.	Provider	S.E.	Elect. Off.	S.E.	Manage.	S.E.	Bus. Owner.	S.E.	Lab. Force	S.E.	Earnings	S.E.	Rel. Poverty	S.E.
**Fixed Part**																		
**CONS**	-1.165	0.032	-1.168	0.032	-1.178	0.031	-1.168	0.032	-1.164	0.032	-1.166	0.032	-1.162	0.032	-1.168	0.031	-1.164	0.032
**Age**	0.035	0.001	0.035	0.001	0.035	0.001	0.035	0.001	0.035	0.001	0.035	0.001	0.035	0.001	0.035	0.001	0.035	0.001
**Equiv. Income**	-0.474	0.013	-0.474	0.013	-0.474	0.013	-0.475	0.013	-0.475	0.013	-0.475	0.013	-0.475	0.013	-0.474	0.013	-0.475	0.013
**Education 2**	-0.682	0.025	-0.682	0.025	-0.681	0.025	-0.682	0.025	-0.682	0.025	-0.682	0.025	-0.682	0.025	-0.682	0.025	-0.682	0.025
**Education 3**	-0.912	0.027	-0.912	0.027	-0.911	0.027	-0.912	0.027	-0.913	0.027	-0.912	0.027	-0.913	0.027	-0.913	0.027	-0.913	0.027
**Education 4**	-1.344	0.029	-1.344	0.029	-1.342	0.029	-1.344	0.029	-1.345	0.029	-1.344	0.029	-1.345	0.029	-1.344	0.029	-1.344	0.029
**Race/Ethnicity 2**	0.148	0.035	0.146	0.035	0.146	0.034	0.147	0.035	0.148	0.034	0.147	0.035	0.148	0.034	0.148	0.034	0.147	0.035
**Race/Ethnicity 3**	0.289	0.042	0.292	0.042	0.295	0.042	0.29	0.042	0.288	0.042	0.291	0.042	0.289	0.042	0.292	0.042	0.29	0.042
**Race/Ethnicity 4**	0.541	0.054	0.544	0.054	0.547	0.054	0.542	0.054	0.539	0.054	0.543	0.054	0.539	0.054	0.543	0.054	0.541	0.054
**Race/Ethnicity 5**	0.503	0.034	0.504	0.034	0.507	0.034	0.504	0.034	0.501	0.034	0.504	0.034	0.498	0.034	0.505	0.034	0.503	0.034
**Employment 2**	0.526	0.038	0.526	0.038	0.527	0.038	0.526	0.038	0.525	0.038	0.526	0.038	0.525	0.038	0.526	0.038	0.526	0.038
**Marital 2**	0.322	0.017	0.323	0.017	0.323	0.017	0.322	0.017	0.322	0.017	0.322	0.017	0.323	0.017	0.323	0.017	0.323	0.017
**Gini**	0.117	0.023	0.124	0.023	0.136	0.021	0.116	0.023	0.104	0.025	0.105	0.025	0.101	0.023	0.139	0.024	0.114	0.023
**GDP**	-0.092	0.023	-0.059	0.026	-0.031	0.025	-0.077	0.024	-0.098	0.024	-0.092	0.023	-0.086	0.022	-0.08	0.022	-0.09	0.023
**Higher Education**	-0.015	0.023																
**Reproductive Rights**			0.057	0.026														
**Provider**					0.1	0.026												
**Elected Office**							0.036	0.023										
**Management**									-0.03	0.025								
**Business Ownership**											0.025	0.024						
**Labour Force**													0.045	0.023				
**Earnings**															0.062	0.024		
**Relative Poverty**																	0.009	0.023
**Random Part**																		
**Level: State**																		
**CONS/CONS**	0.021	0.005	0.019	0.005	0.015	0.004	0.02	0.005	0.02	0.005	0.02	0.005	0.019	0.005	0.018	0.004	0.021	0.005
**Level: Individual**																		
**bcons.1/bcons.1**	1	0	1	0	1	0	1	0	1	0	1	0	1	0	1	0	1	0
**Units: States**	50		50		50		50		50		50		50		50		50	
**Units: Individuals**	116594		116594		116594		116594		116594		116594		116594		116594		116594	

Supporting information 1 - Table B: Multilevel logistic regression for SRH (18–64 yrs.)

	Higher Edu.	S.E.	Rep. Rights	S.E.	Provider	S.E.	Elect. Off.	S.E.	Manage.	S.E.	Bus. Owner.	S.E.	Lab. Force	S.E.	Earnings	S.E.	Rel. Poverty	S.E.
**Fixed Part**																		
**CONS**	-1.318	0.038	-1.321	0.038	-1.328	0.037	-1.32	0.038	-1.318	0.038	-1.32	0.038	-1.315	0.037	-1.32	0.038	-1.318	0.038
**Age**	0.051	0.001	0.051	0.001	0.051	0.001	0.051	0.001	0.051	0.001	0.051	0.001	0.051	0.001	0.051	0.001	0.051	0.001
**Equiv. Income**	-0.557	0.016	-0.557	0.016	-0.557	0.016	-0.557	0.016	-0.558	0.016	-0.558	0.016	-0.558	0.016	-0.557	0.016	-0.557	0.016
**Education 2**	-0.81	0.032	-0.81	0.032	-0.81	0.032	-0.81	0.032	-0.81	0.032	-0.81	0.032	-0.81	0.032	-0.81	0.032	-0.81	0.032
**Education 3**	-1.094	0.034	-1.094	0.034	-1.093	0.034	-1.094	0.034	-1.095	0.034	-1.094	0.034	-1.096	0.034	-1.094	0.034	-1.094	0.034
**Education 4**	-1.556	0.037	-1.555	0.037	-1.554	0.037	-1.555	0.037	-1.556	0.037	-1.555	0.037	-1.557	0.037	-1.555	0.037	-1.556	0.037
**Race/Ethnicity 2**	0.104	0.04	0.102	0.04	0.102	0.04	0.103	0.04	0.105	0.04	0.103	0.04	0.105	0.04	0.105	0.04	0.104	0.04
**Race/Ethnicity 3**	0.32	0.049	0.322	0.049	0.324	0.048	0.32	0.049	0.318	0.049	0.322	0.049	0.317	0.048	0.322	0.049	0.32	0.049
**Race/Ethnicity 4**	0.544	0.063	0.547	0.063	0.55	0.063	0.545	0.063	0.541	0.063	0.547	0.063	0.541	0.063	0.546	0.063	0.545	0.063
**Race/Ethnicity 5**	0.541	0.038	0.542	0.038	0.546	0.038	0.542	0.038	0.538	0.038	0.544	0.038	0.531	0.038	0.543	0.038	0.541	0.038
**Employment 2**	0.485	0.04	0.485	0.04	0.486	0.04	0.485	0.04	0.484	0.04	0.484	0.04	0.483	0.04	0.485	0.04	0.485	0.04
**Marital 2**	0.386	0.021	0.386	0.021	0.386	0.021	0.386	0.021	0.385	0.021	0.386	0.021	0.386	0.021	0.386	0.021	0.386	0.021
**Gini**	0.114	0.024	0.12	0.024	0.129	0.024	0.114	0.024	0.1	0.026	0.102	0.026	0.094	0.024	0.13	0.026	0.113	0.024
**GDP**	-0.085	0.024	-0.064	0.029	-0.041	0.029	-0.076	0.026	-0.095	0.025	-0.087	0.024	-0.079	0.023	-0.078	0.024	-0.085	0.024
**Higher Education**	-0.002	0.024																
**Reproductive Rights**			0.038	0.028														
**Provider**					0.073	0.029												
**Elected Office**							0.025	0.025										
**Management**									-0.037	0.027								
**Business Ownership**											0.027	0.026						
**Labour Force**													0.063	0.024				
**Earnings**															0.042	0.026		
**Relative Poverty**																	0.006	0.024
**Random Part**																		
**Level: State**																		
**CONS/CONS**	0.021	0.006	0.021	0.005	0.018	0.005	0.021	0.005	0.02	0.005	0.021	0.005	0.018	0.005	0.02	0.005	0.021	0.006
**Level: Individual**																		
**bcons.1/bcons.1**	1	0	1	0	1	0	1	0	1	0	1	0	1	0	1	0	1	0
**Units: States**	50		50		50		50		50		50		50		50		50	
**Units: Individuals**	91542		91542		91542		91542		91542		91542		91542		91542		91542	

Supporting information 1 - Table C: Multilevel logistic regression for SRH (65+ yrs.)

	Higher Edu.	S.E.	Rep. Rights	S.E.	Provider	S.E.	Elect. Off.	S.E.	Manage.	S.E.	Bus. Owner.	S.E.	Lab. Force	S.E.	Earnings	S.E.	Rel. Poverty	S.E.
**Fixed Part**																		
**CONS**	-0.446	0.043	-0.452	0.042	-0.467	0.041	-0.452	0.042	-0.444	0.043	-0.446	0.043	-0.443	0.043	-0.45	0.042	-0.447	0.043
**Age**	0.022	0.002	0.022	0.002	0.022	0.002	0.022	0.002	0.022	0.002	0.022	0.002	0.022	0.002	0.022	0.002	0.022	0.002
**Equiv. Income**	-0.342	0.023	-0.341	0.023	-0.341	0.023	-0.342	0.023	-0.342	0.023	-0.342	0.023	-0.342	0.023	-0.341	0.023	-0.342	0.023
**Education 2**	-0.584	0.042	-0.584	0.042	-0.58	0.042	-0.583	0.042	-0.584	0.042	-0.584	0.042	-0.584	0.042	-0.584	0.042	-0.584	0.042
**Education 3**	-0.721	0.046	-0.722	0.046	-0.714	0.046	-0.72	0.046	-0.722	0.046	-0.722	0.046	-0.724	0.046	-0.722	0.046	-0.722	0.046
**Education 4**	-1.103	0.047	-1.101	0.047	-1.094	0.047	-1.101	0.047	-1.104	0.047	-1.103	0.047	-1.105	0.047	-1.103	0.047	-1.103	0.047
**Race/Ethnicity 2**	0.218	0.07	0.21	0.07	0.212	0.069	0.214	0.07	0.218	0.07	0.216	0.07	0.217	0.07	0.218	0.07	0.214	0.07
**Race/Ethnicity 3**	0.217	0.086	0.236	0.085	0.244	0.085	0.217	0.085	0.222	0.086	0.224	0.086	0.22	0.085	0.226	0.085	0.222	0.085
**Race/Ethnicity 4**	0.453	0.105	0.461	0.105	0.469	0.105	0.455	0.105	0.454	0.105	0.456	0.105	0.452	0.105	0.457	0.105	0.455	0.105
**Race/Ethnicity 5**	0.265	0.083	0.268	0.083	0.279	0.083	0.268	0.083	0.264	0.084	0.267	0.084	0.261	0.084	0.272	0.083	0.268	0.084
**Employment 2**	0.208	0.16	0.205	0.16	0.21	0.16	0.205	0.16	0.207	0.16	0.206	0.16	0.207	0.16	0.208	0.16	0.206	0.16
**Marital 2**	0.254	0.031	0.255	0.031	0.255	0.031	0.254	0.031	0.254	0.031	0.254	0.031	0.254	0.031	0.254	0.031	0.254	0.031
**Gini**	0.116	0.027	0.129	0.026	0.141	0.023	0.112	0.026	0.118	0.03	0.106	0.029	0.11	0.029	0.154	0.028	0.113	0.027
**GDP**	-0.094	0.027	-0.041	0.03	-0.004	0.028	-0.071	0.027	-0.091	0.027	-0.095	0.027	-0.091	0.027	-0.079	0.025	-0.094	0.026
**Higher Education**	-0.019	0.027																
**Reproductive Rights**			0.089	0.029														
**Provider**					0.139	0.027												
**Elected Office**							0.057	0.025										
**Management**									0.009	0.03								
**Business Ownership**											0.019	0.027						
**Labour Force**													0.013	0.028				
**Earnings**															0.094	0.027		
**Relative Poverty**																	0.032	0.027
**Random Part**																		
**Level: State**																		
**CONS/CONS**	0.018	0.006	0.015	0.005	0.009	0.004	0.016	0.006	0.019	0.006	0.019	0.006	0.019	0.006	0.014	0.005	0.018	0.006
**Level: Individual**																		
**bcons.1/bcons.1**	1	0	1	0	1	0	1	0	1	0	1	0	1	0	1	0	1	0
**Units: States**	50		50		50		50		50		50		50		50		50	
**Units: Individuals**	25052		25052		25052		25052		25052		25052		25052		25052		25052	
