# Supplementary material for: Is gender inequity a risk factor for men reporting poorer self-rated health in the United States?
Source: PLoS One. 2018 Jul 17;13(7):e0200332. doi: 10.1371/journal.pone.0200332 (PMC6049919; doi:10.1371/journal.pone.0200332)
Supplement: S2 File — (PDF) [file pone.0200332.s002.pdf]

Supporting information 2 - Table A: Sensitivity testing with income in original categories (18+ yrs.)

	Higher Edu.	S.E.	Rep. Rights	S.E.	Provider	S.E.	Elect. Off.	S.E.	Manage.	S.E.	Bus. Owner.	S.E.	Lab. Force	S.E.	Earnings	S.E.	Rel. Poverty	S.E.
**Fixed Part**																		
**CONS**	0.224	0.045	0.221	0.044	0.211	0.044	0.221	0.044	0.225	0.044	0.224	0.044	0.227	0.044	0.221	0.044	0.224	0.044
**Age**	0.031	0.001	0.031	0.001	0.031	0.001	0.031	0.001	0.031	0.001	0.031	0.001	0.031	0.001	0.031	0.001	0.031	0.001
**Income 2**	-0.238	0.043	-0.238	0.043	-0.237	0.043	-0.238	0.043	-0.238	0.043	-0.238	0.043	-0.238	0.043	-0.238	0.043	-0.238	0.043
**Income 3**	-0.599	0.041	-0.599	0.041	-0.599	0.041	-0.599	0.041	-0.599	0.041	-0.599	0.041	-0.599	0.041	-0.599	0.041	-0.599	0.041
**Income 4**	-1.002	0.04	-1.002	0.04	-1.002	0.04	-1.002	0.04	-1.002	0.04	-1.002	0.04	-1.002	0.04	-1.002	0.04	-1.002	0.04
**Income 5**	-1.292	0.039	-1.292	0.039	-1.292	0.039	-1.292	0.039	-1.292	0.039	-1.292	0.039	-1.292	0.039	-1.292	0.039	-1.292	0.039
**Income 6**	-1.578	0.04	-1.578	0.04	-1.578	0.04	-1.578	0.04	-1.578	0.04	-1.578	0.04	-1.578	0.04	-1.578	0.04	-1.578	0.04
**Income 7**	-1.923	0.042	-1.923	0.042	-1.922	0.042	-1.923	0.042	-1.923	0.042	-1.923	0.042	-1.923	0.042	-1.923	0.042	-1.923	0.042
**Income 8**	-2.255	0.044	-2.255	0.044	-2.254	0.044	-2.256	0.044	-2.256	0.044	-2.255	0.044	-2.255	0.044	-2.254	0.044	-2.255	0.044
**Education 2**	-0.46	0.026	-0.46	0.026	-0.46	0.026	-0.46	0.026	-0.461	0.026	-0.46	0.026	-0.461	0.026	-0.461	0.026	-0.46	0.026
**Education 3**	-0.603	0.029	-0.603	0.029	-0.602	0.029	-0.603	0.029	-0.604	0.029	-0.603	0.029	-0.604	0.029	-0.603	0.029	-0.603	0.029
**Education 4**	-0.968	0.031	-0.968	0.031	-0.966	0.031	-0.968	0.031	-0.969	0.031	-0.968	0.031	-0.969	0.031	-0.968	0.031	-0.968	0.031
**Race/Ethnicity 2**	0.032	0.036	0.03	0.036	0.03	0.036	0.031	0.036	0.032	0.036	0.031	0.036	0.032	0.036	0.032	0.036	0.031	0.036
**Race/Ethnicity 3**	0.179	0.043	0.182	0.043	0.184	0.043	0.179	0.043	0.177	0.043	0.181	0.043	0.178	0.043	0.181	0.043	0.18	0.043
**Race/Ethnicity 4**	0.457	0.056	0.46	0.056	0.463	0.056	0.458	0.056	0.454	0.056	0.459	0.056	0.455	0.056	0.459	0.056	0.457	0.056
**Race/Ethnicity 5**	0.326	0.035	0.327	0.035	0.33	0.035	0.327	0.035	0.323	0.035	0.327	0.035	0.32	0.035	0.328	0.035	0.326	0.035
**Employment 2**	0.186	0.04	0.186	0.04	0.187	0.04	0.186	0.04	0.185	0.04	0.186	0.04	0.185	0.04	0.186	0.04	0.186	0.04
**Marital 2**	-0.006	0.019	-0.006	0.019	-0.006	0.019	-0.006	0.019	-0.007	0.019	-0.006	0.019	-0.006	0.019	-0.006	0.019	-0.006	0.019
**Gini**	0.108	0.022	0.115	0.022	0.126	0.02	0.107	0.021	0.094	0.023	0.097	0.024	0.093	0.022	0.13	0.023	0.105	0.022
**GDP**	-0.07	0.022	-0.041	0.025	-0.014	0.025	-0.056	0.023	-0.078	0.022	-0.071	0.022	-0.065	0.021	-0.06	0.021	-0.069	0.022
**Higher Education**	-0.008	0.022																
**Reproductive Rights**			0.052	0.025														
**Provider**					0.093	0.025												
**Elected Office**							0.036	0.022										
**Management**									-0.034	0.024								
**Business Ownership**											0.023	0.023						
**Labour Force**													0.044	0.022				
**Earnings**															0.058	0.023		
**Relative Poverty**																	0.014	0.022
**Random Part**																		
**Level: State**																		
**CONS/CONS**	0.018	0.004	0.017	0.004	0.013	0.004	0.017	0.004	0.017	0.004	0.018	0.004	0.016	0.004	0.016	0.004	0.018	0.004
**Level: Individual**																		
**bcons.1/bcons.1**	1	0	1	0	1	0	1	0	1	0	1	0	1	0	1	0	1	0
**Units: State**	50		50		50		50		50		50		50		50		50	
**Units: Individual**	116594		116594		116594		116594		116594		116594		116594		116594		116594	

Supporting information 2 - Table B: Sensitivity testing with household median income instead of GDP (18+ yrs.)

	Higher Edu.	S.E.	Rep. Rights	S.E.	Provider	S.E.	Elect. Off.	S.E.	Manage.	S.E.	Bus. Owner.	S.E.	Lab. Force	S.E.	Earnings	S.E.	Rel. Poverty	S.E.
**Fixed Part**																		
**CONS**	-1.169	0.032	-1.172	0.031	-1.179	0.031	-1.174	0.031	-1.17	0.032	-1.173	0.032	-1.167	0.031	-1.171	0.031	-1.17	0.032
**Age**	0.035	0.001	0.035	0.001	0.035	0.001	0.035	0.001	0.035	0.001	0.035	0.001	0.035	0.001	0.035	0.001	0.035	0.001
**Equiv. Income**	-0.474	0.013	-0.474	0.013	-0.474	0.013	-0.474	0.013	-0.474	0.013	-0.474	0.013	-0.474	0.013	-0.474	0.013	-0.474	0.013
**Education 2**	-0.682	0.025	-0.682	0.025	-0.681	0.025	-0.682	0.025	-0.682	0.025	-0.682	0.025	-0.682	0.025	-0.682	0.025	-0.682	0.025
**Education 3**	-0.912	0.027	-0.912	0.027	-0.911	0.027	-0.912	0.027	-0.913	0.027	-0.911	0.027	-0.913	0.027	-0.912	0.027	-0.912	0.027
**Education 4**	-1.344	0.029	-1.343	0.029	-1.342	0.029	-1.343	0.029	-1.344	0.029	-1.343	0.029	-1.344	0.029	-1.344	0.029	-1.343	0.029
**Race/Ethnicity 2**	0.149	0.034	0.147	0.035	0.146	0.034	0.148	0.034	0.15	0.034	0.148	0.034	0.15	0.034	0.149	0.034	0.149	0.035
**Race/Ethnicity 3**	0.291	0.042	0.293	0.042	0.295	0.042	0.29	0.042	0.289	0.042	0.293	0.042	0.289	0.042	0.292	0.042	0.291	0.042
**Race/Ethnicity 4**	0.543	0.054	0.545	0.054	0.547	0.054	0.543	0.054	0.541	0.054	0.546	0.054	0.54	0.054	0.544	0.054	0.543	0.054
**Race/Ethnicity 5**	0.502	0.034	0.504	0.034	0.507	0.034	0.504	0.034	0.501	0.034	0.506	0.034	0.497	0.034	0.504	0.034	0.504	0.034
**Employment 2**	0.526	0.038	0.526	0.038	0.527	0.038	0.526	0.038	0.525	0.038	0.526	0.038	0.525	0.038	0.526	0.038	0.526	0.038
**Marital 2**	0.322	0.017	0.323	0.017	0.323	0.017	0.322	0.017	0.322	0.017	0.322	0.017	0.323	0.017	0.322	0.017	0.322	0.017
**Gini**	0.085	0.023	0.102	0.024	0.126	0.025	0.089	0.023	0.071	0.025	0.068	0.025	0.07	0.023	0.107	0.026	0.084	0.023
**Median State HH Inc.**	-0.103	0.023	-0.074	0.027	-0.032	0.03	-0.092	0.023	-0.112	0.024	-0.11	0.023	-0.102	0.022	-0.088	0.024	-0.103	0.023
**Higher Education**	0.004	0.022																
**Reproductive Rights**			0.049	0.026														
**Provider**					0.096	0.03												
**Elected Office**							0.041	0.022										
**Management**									-0.033	0.025								
**Business Ownership**											0.037	0.024						
**Labour Force**													0.052	0.022				
**Earnings**															0.044	0.025		
**Relative Poverty**																	0.014	0.022
**Random Part**																		
**Level: State**																		
**CONS/CONS**	0.019	0.005	0.018	0.004	0.015	0.004	0.018	0.004	0.019	0.005	0.018	0.004	0.017	0.004	0.018	0.004	0.019	0.005
**Level: Individual**																		
**bcons.1/bcons.1**	1	0	1	0	1	0	1	0	1	0	1	0	1	0	1	0	1	0
**Units: States**	50		50		50		50		50		50		50		50		50	
**Units: Individuals**	116594		116594		116594		116594		116594		116594		116594		116594		116594	

Supporting information 2 - Table C: Sensitivity testing comparing PQL2 and MCMC estimator for reproductive rights model (18+ yrs.)

	Rep. Rights PQL2	S.E.	Rep. Rights MCMC	S.E.
**Fixed Part**				
CONS	-1.168	0.032	-1.169	0.033
Age	0.035	0.001	0.035	0.001
Equiv. Income	-0.474	0.013	-0.474	0.013
Education 2	-0.682	0.025	-0.681	0.025
Education 3	-0.912	0.027	-0.912	0.027
Education 4	-1.344	0.029	-1.343	0.029
Race/Ethnicity 2	0.146	0.035	0.145	0.035
Race/Ethnicity 3	0.292	0.042	0.292	0.042
Race/Ethnicity 4	0.544	0.054	0.544	0.054
Race/Ethnicity 5	0.504	0.034	0.505	0.034
Employment 2	0.526	0.038	0.526	0.038
Marital 2	0.323	0.017	0.323	0.017
Gini	0.124	0.023	0.124	0.025
GDP	-0.059	0.026	-0.058	0.029
Reproductive Rights	0.057	0.026	0.058	0.028
**Random Part**				
Level: State				
CONS/CONS	0.019	0.005	0.022	0.006
Level: Individual				
bcons.1/bcons.1	1	0	1	0
-2*loglikelihood:				
DIC:			92267.317	
pD:			53.645	
Units: States	50		50	
Units: Individuals	116594		116594	

Supporting information 2 - Table D: Missing data imputation models for 65+ (yrs.) reproductive rights model and 40–49 (yrs.) labour force model

65+ Age Group

	Pre-imputation	S.E.	Post-imputation	S.E.
Fixed Part				
CONS	-2.137	0.174	-2.048	0.159
Age	0.022	0.002	0.021	0.002
Equiv. Inc. (missing)	-0.341	0.023	-0.267	0.021
Education 2	-0.584	0.042	-0.56	0.038
Education 3	-0.722	0.046	-0.728	0.043
Education 4	-1.101	0.047	-1.133	0.044
Race/Ethnicity 2	0.21	0.07	0.186	0.064
Race/Ethnicity 3	0.236	0.085	0.204	0.079
Race/Ethnicity 4	0.461	0.105	0.449	0.098
Race/Ethnicity 5	0.268	0.083	0.29	0.078
Employment 2	0.205	0.16	0.197	0.148
Marital 2	0.255	0.031	0.247	0.028
Gini	0.129	0.026	0.125	0.025
GDP	-0.041	0.03	-0.041	0.029
Reproductive Rights	0.089	0.029	0.089	0.028
Random Part				
Level: State				
CONS/CONS	0.015	0.005	0.015	0.005
Level: Individual				
bcons.1/bcons.1	1	0	1	0
Units: State	50		50	
Units: Individual	25052		25052	

40-49 Age Group

	Pre-imputation	S.E.	Post-imputation	S.E.
Fixed Part				
CONS	-4.493	0.331	-4.507	0.318
Age	0.073	0.007	0.075	0.007
Equiv. Inc. (missing)	-0.659	0.036	-0.55	0.034
Education 2	-0.994	0.06	-1.016	0.057
Education 3	-1.24	0.066	-1.281	0.063
Education 4	-1.645	0.073	-1.728	0.071
Race/Ethnicity 2	0.062	0.078	0.071	0.075
Race/Ethnicity 3	0.369	0.092	0.443	0.086
Race/Ethnicity 4	0.453	0.124	0.467	0.119
Race/Ethnicity 5	0.34	0.076	0.371	0.073
Employment 2	0.499	0.075	0.499	0.072
Marital 2	0.403	0.042	0.42	0.041
Gini	0.08	0.032	0.082	0.032
GDP	-0.086	0.03	-0.085	0.03
Labour Force	0.07	0.031	0.069	0.032
Random Part				
Level: State				
CONS/CONS	0.016	0.008	0.019	0.008
Level: Individual				
bcons.1/bcons.1	1	0	1	0
Units: State	50		50	
Units: Individual	24639		24639	
