# Supplementary material for: Is gender inequity a risk factor for men reporting poorer self-rated health in the United States?
Source: PLoS One. 2018 Jul 17;13(7):e0200332. doi: 10.1371/journal.pone.0200332 (PMC6049919; doi:10.1371/journal.pone.0200332)
Supplement: S3 File — (PDF) [file pone.0200332.s003.pdf]

Notes on data preparation

Kavanagh SA, Shelley JM, Stevenson C. Is gender inequity a risk factor for men reporting poorer self-rated health in the United States? PLOSONE, 2018

All data are freely available. The following notes provide instructions to recreate the data for analysis.

Individual-level variables

Data for self-rated health and individual-level covariates were derived from the Behavioral Risk Factor Surveillance System 2005 data set.
https://www.cdc.gov/brfss/index.html

State-level variables

Higher education
Source: American Fact Finder at US Census Bureau website
http://factfinder2.census.gov/faces/nav/jsf/pages/index.xhtml
File downloaded as Excel file (B15002) 2005
Calculation: Bachelors/Masters/Professional/Doctorate summed for each sex and divided by total population for sex, then male divided by female * 100

Reproductive rights
Source: Reproductive Rights Composite Index from The Status of Women in the States (SWS) report 2004 Table 9, p. 43
http://www.iwpr.org/publications/pubs/the-status-of-women-in-the-states

Abortion provider
Source: Reproductive Rights Index from SWS report 2004 Table 9, p. 43
http://www.iwpr.org/publications/pubs/the-status-of-women-in-the-states

Elected office
Source: Women in Elected Office Composite Index taken from 2004 SWS report Table 2, p. 13.
http://www.iwpr.org/publications/pubs/the-status-of-women-in-the-states

Management
Source: American Fact Finder - 2005 American Community Survey
Sex by occupation for the civilian employed population 16 years and over Table B24010
Calculation: Male management occupations divided by male population. Female management occupations divided by female population. Male result divided by female result * 100

From table selected ‘Management Occupations’. Superior to ‘Management, Business and Financial Occupations’ as this may include occupations that do not hold responsibility and power.

Business Ownership
Source: American Fact Finder - Survey of business owners 2002 Table SB0200A1
Calculation: Number of firms with or without paid employees
Male owned divided by (female+equally owned) * 100

Labour Force
Source: American Fact Finder - Employment status - 2005 American Community Survey S2301
Calculation: Population 20 to 64 years: male percentage in labour force divided by female percentage labour force * 100

Earnings
Source: Table retrieved from American Fact Finder website:
S2001-Earnings in the past 12 months (In 2005 inflation-adjusted dollars) 2005 American Community Survey - median earnings FT year round
Calculation: Male divided by female * 100

Gender inequity - relative poverty
Source: American Fact Finder B17001 - 2005: Poverty status in the past 12 months by sex by age
Calculation: Table did not have separate male/female population so sex ratio was taken from Table S0101 - 2005 to calculate absolute populations of male and female. Then population with income in the past 12 months below poverty level divided by total population for male and female. Female % below poverty divided by male % below poverty * 100

Income inequality
Source: Household Income Inequality Measures Based on ACS Data: 2000–2005 paper - US Census Bureau - Gini 2004
https://www.census.gov/content/dam/Census/library/working-papers/2007/demo/ACS-inequality-report-2000-2005_v2.pdf
Table 5: State-level mean household income and selected income inequality measures, 2004

GDP
Source: Real per capita GDP 2004 by state (chained 2009 dollars) from US Department of Commerce - Bureau of Economic Analysis taken from table.
http://www.bea.gov/itable/iTable.cfm?ReqID=70&step=1#reqid=70&step=1&isuri=1

State-level median income
Source: Income, Poverty, and Health Insurance Coverage in the United States: 2004 US Census Bureau P60-229
http://www.census.gov/prod/2005pubs/p60-229.pdf
Table 9. Income of households by state using 2- and 3-year-average medians: 2002 to 2004 (Income in 2004 dollars), p. 23; 3-year average 2002–2004

Notes on calculation of equivalised household income

Calculated from the household income variable; eight categories of household income were provided from the BRFSS data set: less than $10,000; $10,000 to <$15,000; $15,000 to <$20,000; $20,000 to <$25,000; $25,000 to <$35,000; $35,000 to <$50,000; $50,000 to <$75,000; $75,000 or more. To calculate equivalised income a mid-point value was substituted for each of the categories. In the case of the top category a value was calculated utilising a Pareto distribution following [1] - see below. Each of these values was divided by the square root of the total number of persons in each household [2].

Calculation of dollar value for top category for income:

Following paper by Ligon [1]
Calculate v=c-d/b-a=1.354
a=log10 lower bound of category preceding top category=log10(50,000)=
b=log10 lower bound top category=log10(75,000)
c=log10sum of frequencies top two categories (22082+30160)
d=log10 frequency top category (30160)

value=75000*(v/v-1)=286319

Mid-point categories:

INCOME2 Recode_1	less than $10,000	$5000
INCOME2 Recode_2	$10,000 to <$15,000	$12500
INCOME2 Recode_3	$15000 to <$20,000	$17500
INCOME2 Recode_4	$20,000 to <$25,000	$22500
INCOME2 Recode_5	$25,000 to <$35,000	$30000
INCOME2 Recode_6	$35,000 to <$50,000	$42500
INCOME2 Recode_7	$50,000 to <$75,000	$62500
INCOME2 Recode_8	$75,000 or more	$286319

Recoding of variables

RECODING OF VARIABLES		
Variable	Original Code	Recode
Marital	Married	Married/Couple (1)
	Member of an Unmarried Couple	Married/Couple (1)
	Divorced	Unmarried/No Couple (2)
	Widowed	Unmarried/No Couple (2)
	Separated	Unmarried/No Couple (2)
	Never Married	Unmarried/No Couple (2)
Employment	employed for wages	Other (1)
	self-employed	Other (1)
	homemaker	Other (1)
	student	Other (1)
	retired	Other (1)
	unable to work	Other (1)
	out of work	Unemployed (2)

Sensitivity testing: Notes on missing data imputation

MLwiN requires full cases for analysis. For the full data set, approximately 12% of cases were lost due to missing data. The level of missing data for most variables was minimal (<= 1% of cases). However, for the income variable approximately 10% of data were missing. Missing data can lead to biased results. A solution to this issue is to undertake multiple imputation of missing data based on the assumption of missing at random [3]. REALCOM software provides the functionality to undertake multiple imputation in multilevel data sets [3, 4]. Multilevel multiple imputation is, however, computationally intensive. As such, the modelling of missing data for this study posed a challenge given that the models were complex and that the number of cases was large.

To overcome this issue a 'workaround' method was utilised. Smaller age band restricted subsets of data were constructed with missing data only on the income variable to reduce the computational load.

Model 1: 65+ years; this data set had 29,244 individuals nested in 50 states. The full case analysis has 25,052 individuals, representing a loss of approximately 14% of cases due to missing data on the income variable. This was a higher percentage than the 18+ data set where approximately 10% of cases were lost due to missing income data.

Model 2: 40–49 years; this data set had 26,487 men nested in 50 states. The full case analysis had 24,639 individuals representing approximately 7% of cases lost due to missing income data.

The settings for multiple imputation in REALCOM were a burn-in of 1000 iterations, followed by 10,000 updates with 20 imputations [3]. Original estimation models were undertaken with PQL2 estimation.

References

1. Ligon E. The development and use of a consistent income measure for the general social survey. GSS Methodological, Report No. 64; 1994.

2. OECD. What are equivalence scales? [Internet] OECD. [cited 27 April 2018]. Available: http://www.oecd.org/eco/growth/OECD-Note-EquivalenceScales.pdf

3. Carpenter JR, Goldstein H, Kenward MG. REALCOM-IMPUTE software for multilevel multiple imputation with mixed response types. J Stat Softw. 2011; 45. Available: http://www.jstatsoft.org/v45/i05/paper

4. Goldstein, H, Steele, F, Rasbash, J & Charlton, C, 'REALCOM: methodology for realistically complex multilevel modelling'; 2008 [cited 27 April 2018]. Available: http://www.bristol.ac.uk/media-library/sites/cmm/migrated/documents/realcom-training.pdf
